# Novel Genotypes of H9N2 Influenza A Viruses Isolated from Poultry in Pakistan Containing NS Genes Similar to Highly Pathogenic H7N3 and H5N1 Viruses

**DOI:** 10.1371/journal.pone.0005788

**Published:** 2009-06-11

**Authors:** Munir Iqbal, Tahir Yaqub, Kolli Reddy, John W. McCauley

**Affiliations:** 1 Division of Microbiology, Institute for Animal Health, Compton Laboratory, Compton, Newbury, Berkshire, United Kingdom; 2 University Diagnostic Laboratory (UDL), University of Veterinary and Animal Sciences, Lahore, Pakistan; 3 Division of Virology, MRC National Institute for Medical Research, Mill Hill, London, United Kingdom; Mount Sinai School of Medicine, United States of America

## Abstract

The impact of avian influenza caused by H9N2 viruses in Pakistan is now significantly more severe than in previous years. Since all gene segments contribute towards the virulence of avian influenza virus, it was imperative to investigate the molecular features and genetic relationships of H9N2 viruses prevalent in this region. Analysis of the gene sequences of all eight RNA segments from 12 viruses isolated between 2005 and 2008 was undertaken. The hemagglutinin (HA) sequences of all isolates were closely related to H9N2 viruses isolated from Iran between 2004 and 2007 and contained leucine instead of glutamine at position 226 in the receptor binding pocket, a recognised marker for the recognition of sialic acids linked α2–6 to galactose. The neuraminidase (NA) of two isolates contained a unique five residue deletion in the stalk (from residues 80 to 84), a possible indication of greater adaptation of these viruses to the chicken host. The HA, NA, nucleoprotein (NP), and matrix (M) genes showed close identity with H9N2 viruses isolated during 1999 in Pakistan and clustered in the A/Quail/Hong Kong/G1/97 virus lineage. In contrast, the polymerase genes clustered with H9N2 viruses from India, Iran and Dubai. The NS gene segment showed greater genetic diversity and shared a high level of similarity with NS genes from either H5 or H7 subtypes rather than with established H9N2 Eurasian lineages. These results indicate that during recent years the H9N2 viruses have undergone extensive genetic reassortment which has led to the generation of H9N2 viruses of novel genotypes in the Indian sub-continent. The novel genotypes of H9N2 viruses may play a role in the increased problems observed by H9N2 to poultry and reinforce the continued need to monitor H9N2 infections for their zoonotic potential.

## Introduction

Avian influenza viruses naturally circulate in wild aquatic birds as a diverse population having 16 HA and 9 NA antigenic subtypes [1], [2]. When these wild bird-origin viruses are transmitted to new hosts, including domestic gallinaceous poultry, horses, swine and humans, they can undergo adaptive changes leading to the establishment of infection with increased transmissibility and pathogenicity; adaptation to domestic poultry species is seen most frequently [3], [4]. To date, mainly viruses belonging to H5, H7 and H9 subtypes have gained sufficient adaptive molecular changes to become established in domestic poultry and to cause mild to severe disease [5], [6]. These viruses also pose a threat of zoonotic infection [7], [8], [9], [10]. In contrast to H5 and H7 viruses, avian H9 subtypes exist only as low pathogenicity avian influenza (LPAI) viruses. The H9N2 subtype has become prevalent in domestic poultry in many countries in Asia and the Middle East since the late 1990's [5], [11], [12], [13], [14]. Despite being LPAI viruses, these viruses have gained the ability to cause severe respiratory distress accompanied by high morbidity and mortality and a marked reduction in egg production [15], [16]. The frequent heavy losses incurred with H9N2 infection have raised serious concerns for the poultry industry in many countries.

In Pakistan the first H9N2 outbreak in poultry was reported in 1998 [13]. These viruses showed a close relationship with H9N2 viruses circulating in Hong Kong in 1997, and were phylogenetically grouped together within the G1-lineage (Qa/HK/G1/97, HK/1073/99) of H9N2 viruses [17]. Since 1999 the poultry industry in Pakistan has also experienced sporadic infection with H7N3 and H5N1 highly pathogenic avian influenza (HPAI) viruses [18] including the cross-species transmission of H5N1 viruses to humans in late 2007, thought to have led to a family cluster of human cases [19]. In response to the circulation of these viruses in poultry an extensive vaccination programme against H5, H7 and H9 subtype viruses is practised to reduce their impact [20]. The extensive co-circulation of H9N2 viruses with other avian influenza viruses, including highly pathogenic H5N1 and H7N3 subtypes, coupled with extensive vaccination, is likely to generate appropriate conditions for the generation of novel variant and reassortant viruses, possibly with increased epizootic and zoonotic potential.

To analyse the genetic nature of H9N2 viruses in the enzootic region of Pakistan, we sequenced the complete coding regions of all eight segments of twelve H9N2 viruses isolated between May 2005 and March 2008 from poultry flocks in the Punjab and the North West Frontier Province (NWFP) of Pakistan. The results showed that these H9N2 viruses had changed considerably compared with previous H9N2 viruses. The eight gene segments of these viruses no longer clustered in a single established Eurasian H9N2 virus sublineage but there was evidence for complex reassortment of genes from viruses belonging to H9N2 (G1-lineage) HPAI H5N1 (Z-genotype), and HPAI H7N3 viruses. Therefore, these viruses represent novel genotypes of H9N2 viruses, the potential consequences of which should not be overlooked.

## Materials and Methods

### Virus isolation and propagation

The twelve viruses under study (UDL viruses) were isolated at the University Diagnostic Laboratory (UDL) Lahore, from farm outbreaks in the Punjab and the NWFP of Pakistan (Table S1). Viruses were propagated in 10-day old embryonated hens' eggs and initial subtype identification was performed using standard hemagglutination-inhibition (HI) assays and neuraminidase inhibition assays [21] using a panel of reference antisera and reference antigens obtained from the OIE Avian Influenza Reference Laboratory (VLA Weybridge-UK).

### Intravenous pathogenicity testing

Intravenous pathogenicity testing was carried out using standard methods [22] with groups of 10, 6-week old chicks infected with virus diluted in physiological saline. Infected birds were examined for disease signs daily and the intravenous pathogenicity index (IVPI) was recorded.

### Nucleotide sequence analysis

Viral RNA was extracted from allantoic fluid using the High Pure RNA Extraction Kit (Roche Diagnostics) and cDNA was prepared using an influenza universal oligonucleotide primer, 5′AGCAAAAGCAGG-3′ with the Verso™ cDNA Kit (Thermo Scientific). PCR amplification was performed using Pfu Ultra II fusion HS DNA polymerase (Stratagene) and specific primers for the eight gene segments. For HA, both specific and universal primers [23] were used, with slight modification, to amplify HA sequences of any other subtype (H1–H16) that may be present in the sample (primer sequences are available on request). In most cases, PCR products were gel purified using a gel extraction kit (QIA quick, Qiagen) and full-length gene segments were cloned into pCR-Blunt (Zero Blunt PCR Cloning Kit, Invitrogen). The exceptions were that the PB2, PB1 and PA genes were amplified in two overlapping amplicons. A minimum of five independent clones from each gene segment for each virus were sequenced using a commercial sequencing service (*GATC Biotech*, *Constance*, *Germany*). Five virus samples that contained the NS gene homologous to H5N1 viruses and the two samples (Ck/Pak/UDL-01/06 and Ck/Pak/UDL-03/08) that contained the NS gene homologous to H7N3 were re-examined in greater detail. The HA genes of these seven virus samples were subjected to in-depth sequence analysis to exclude the possibility that the samples were from mixed infections with H9N2 viruses and H5N1 or H7N3 viruses. Sequence analyses of the HA genes were performed by sequencing a minimum of 60 clones per sample that was calculated to detect with 95% probability any variant sequences at a level of 5% within the virus sample.

Sequence data were analysed using the Staden package (pregap4 v1.5 and gap4 v1.0). Blast homology searches (http://www.ebi.ac.uk/Tools/fasta33/nucleotide.html) were used to retrieve the top fifty homologous sequences to the sequenced gene from public sequence databases. Multiple nucleotide and amino acid sequence alignments for all eight gene segments were performed using ClustalX (version 1.83). Unrooted phylogenetic trees were generated from nucleotide sequences based on the complete open reading frame of all eight gene segments using minimum evolution analysis with maximum composite likelihood and the Tamura-Nei model [24] with 1000 bootstrapping replications in Molecular Evolutionary Genetics Analysis (MEGA, version 4. 1 beta). The nucleotide sequences obtained in this study have been submitted to the GenBank database and are available under accession numbers (CY038391 to CY038486).

## Results and Discussion

### Phylogenetic analysis

**Figure 1:**
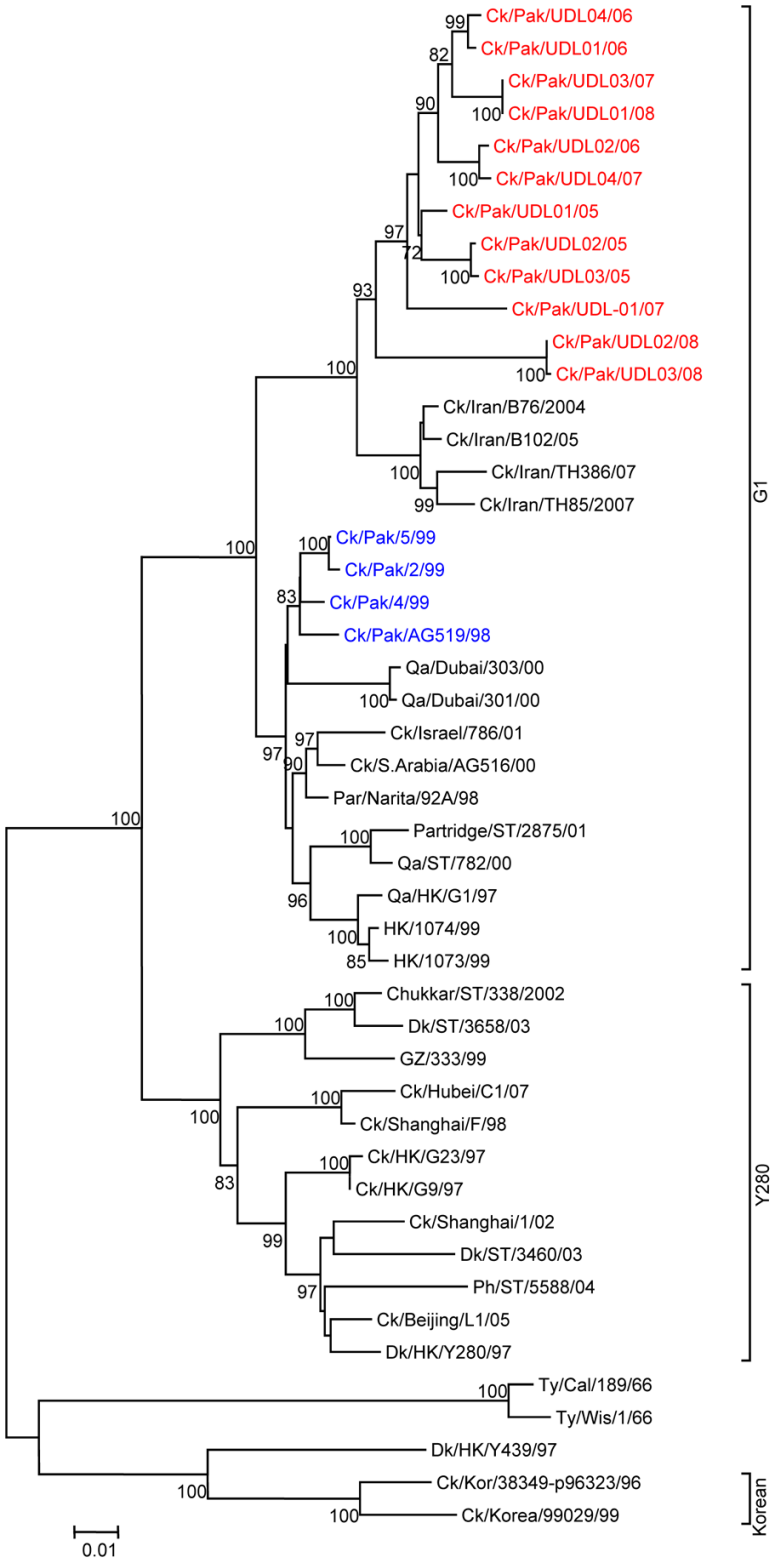
Phylogenetic relationships of HA genes of H9N2 avian influenza viruses isolated from Pakistan between 2005 and 2008. The Phylogenetic tree was generated using minimum evolution analysis with maximum composite likelihood using the Tamura-Nei model [24] with MEGA (version 4.1 beta). Numbers below branches indicate bootstrap value percentages from 1000 replicates, bootstrap values >70% are shown. Analysis was based on complete open reading frames of all gene segments. The scale bar represents the distance unit between sequence pairs. Bhgs, bar headed goose, GF, guinea fowl; Ph, pheasant, Ck, chicken; Dk, duck; Qa, quail; Ty, turkey; Par, parakeet, Sw, swine; Pak, Pakistan; RP, Rawalpindi; Afg, Afghanistan; HK, Hong Kong; ST, Shantou; VN, Vietnam; Wis, Wisconsin; Cal, California; Kor, Korea; GZ, Guangzhou; Rus, Russia; UP, Uttar Pradesh. The viruses characterised in this report are indicated as red. The sequences of H9N2 and H7N3 viruses previously isolated from Pakistan are indicated blue and pink respectively.

**Figure 2:**
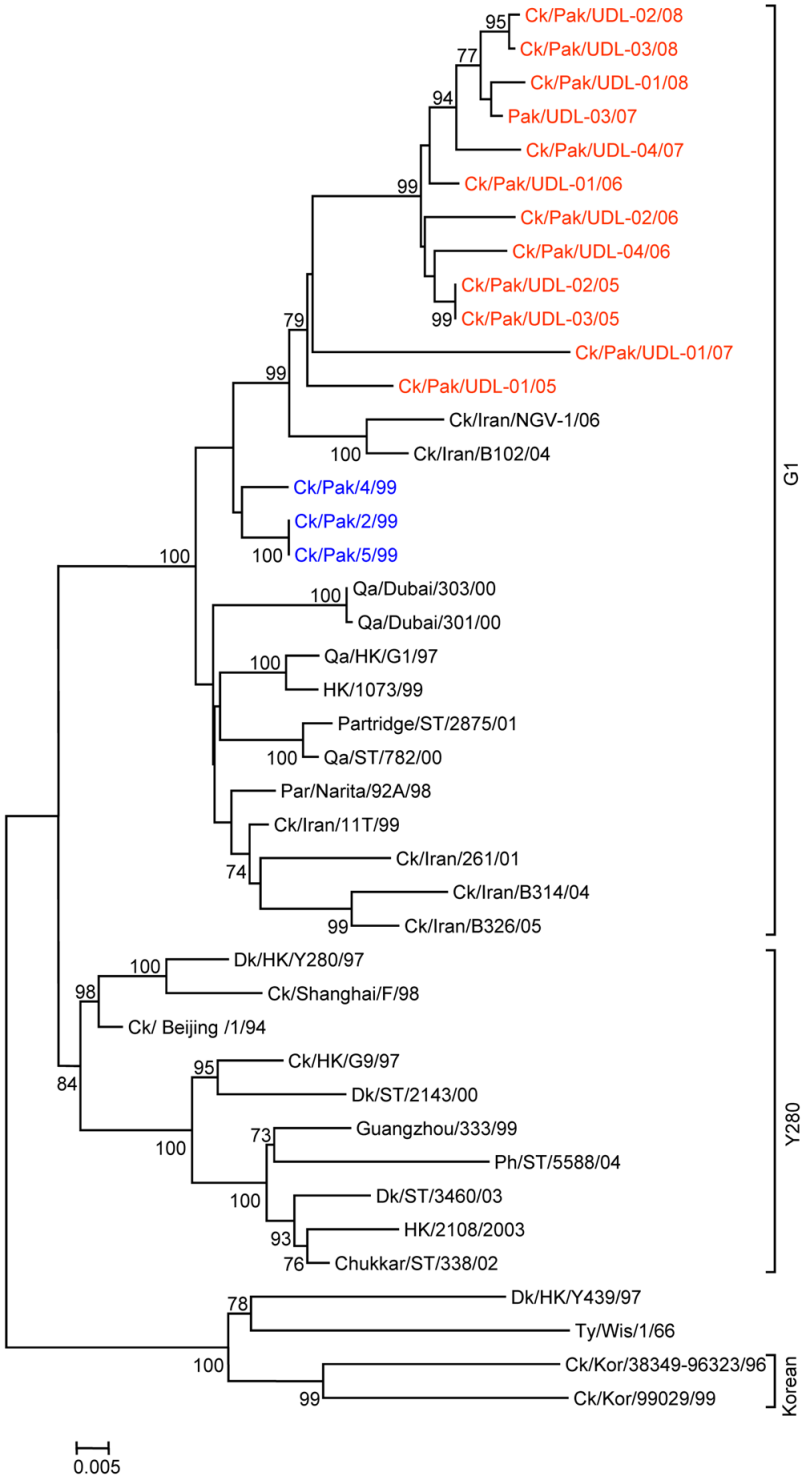
Phylogenetic relationships of the NA genes. The phylogenetic methods and abbreviations were as described for figure 1.

**Figure 3:**
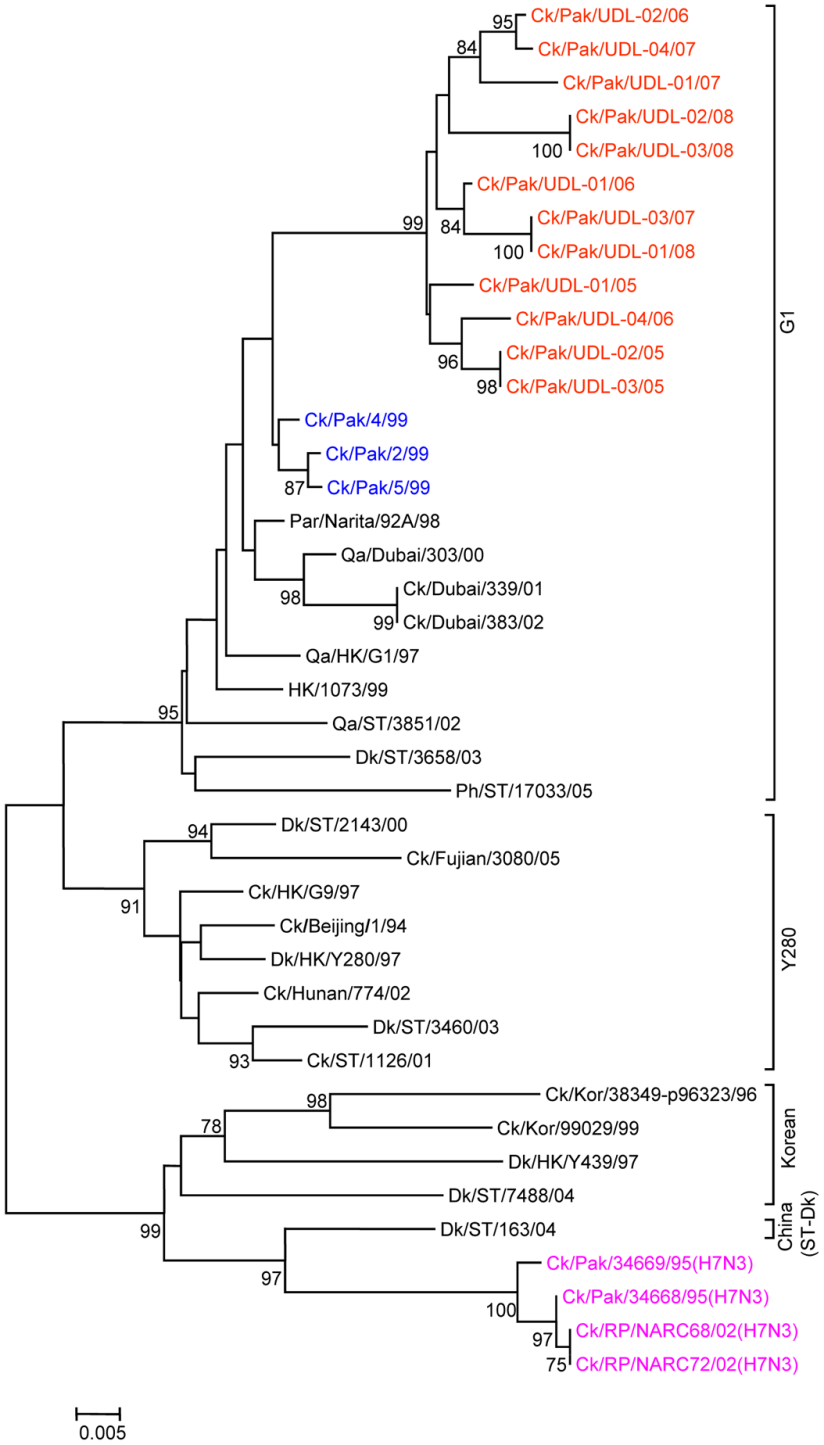
Phylogenetic relationships of the M genes. The phylogenetic methods and abbreviations were as described for figure 1.

**Figure 4:**
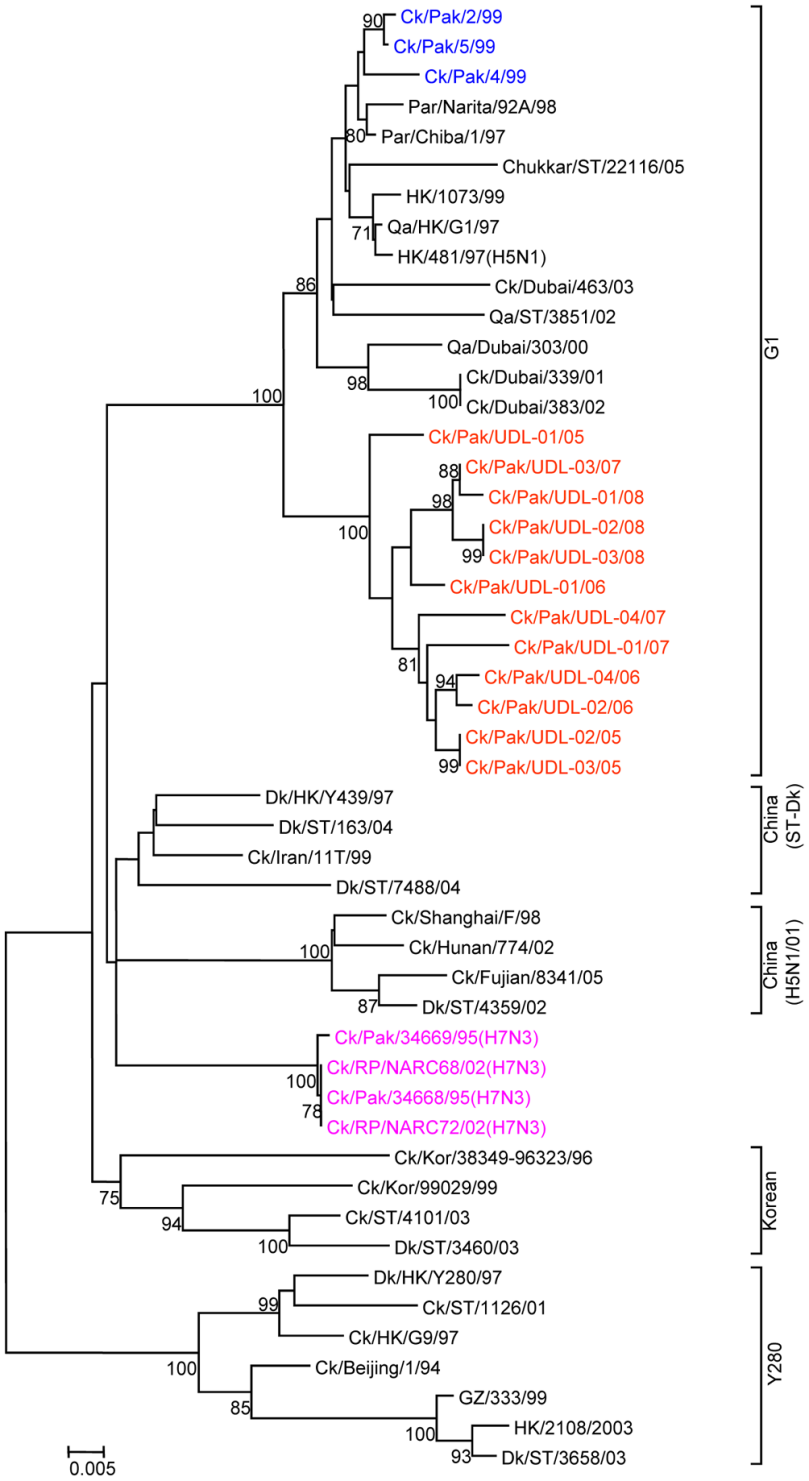
Phylogenetic relationships of the NP genes. The phylogenetic methods and abbreviations were as described for figure 1.

**Figure 5:**
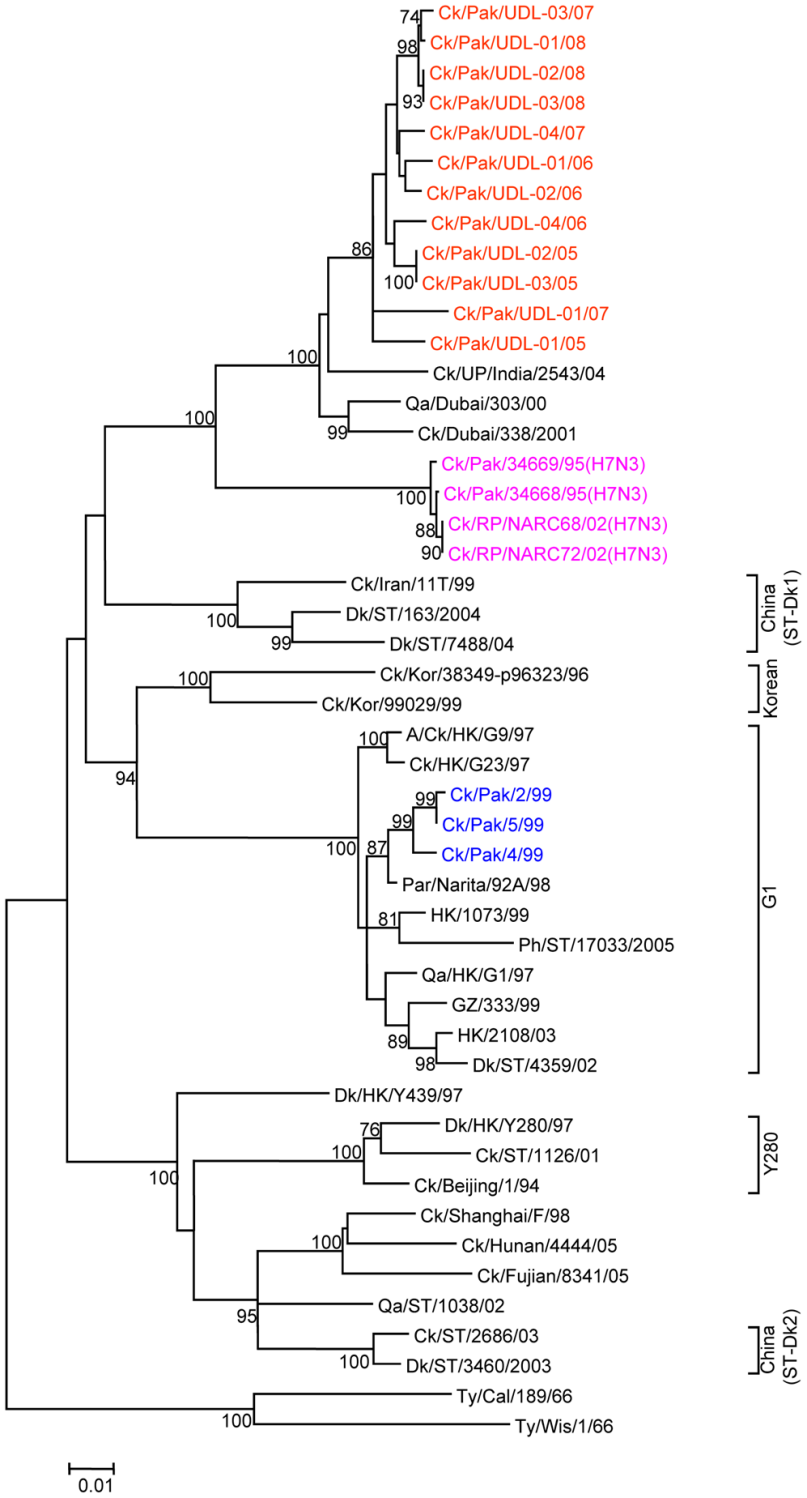
Phylogenetic relationships of the PB2 genes. The phylogenetic methods and abbreviations were as described for figure 1.

**Figure 6:**
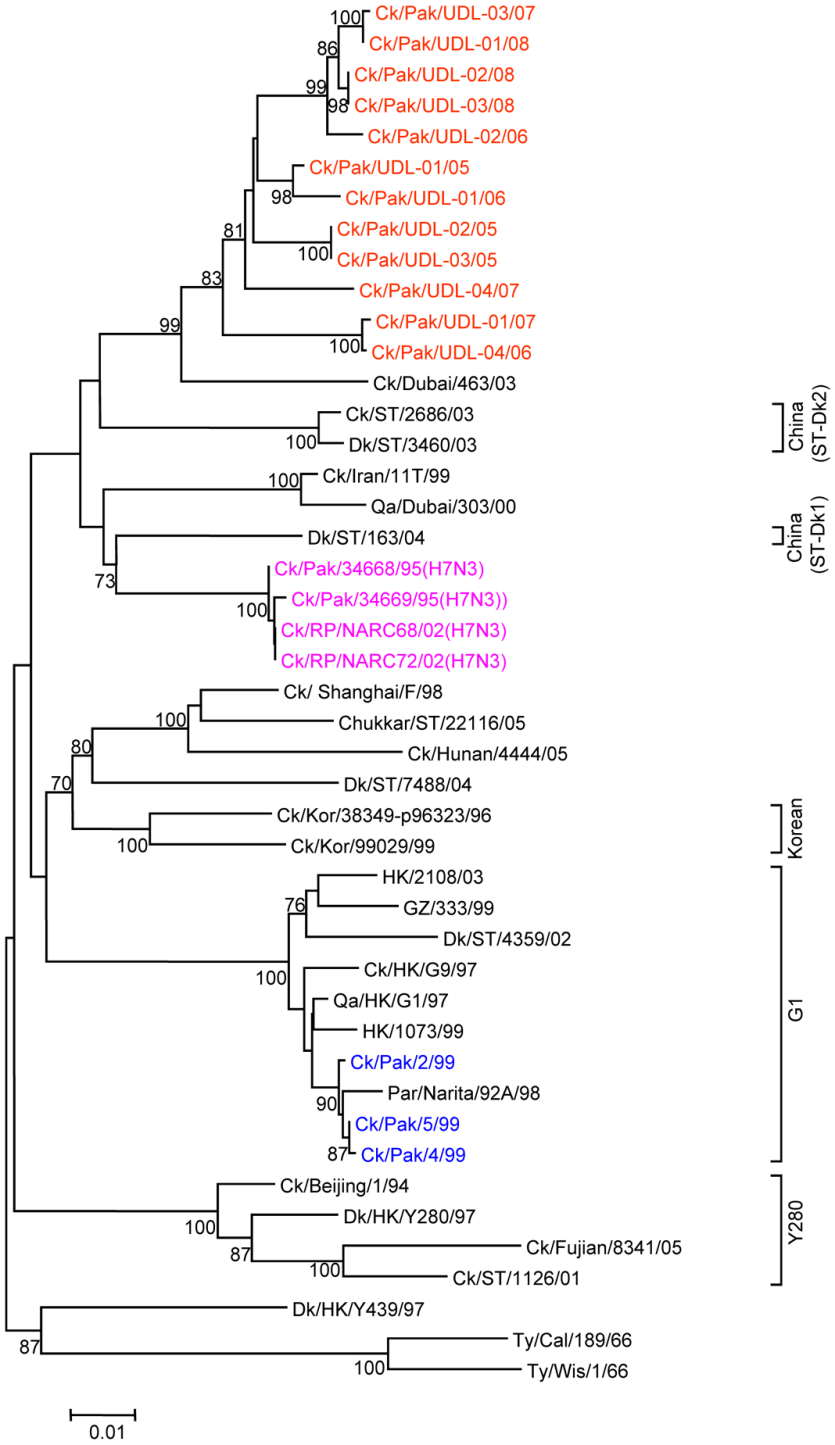
Phylogenetic relationships of the PB1 genes. The phylogenetic methods and abbreviations were as described for figure 1.

**Figure 7:**
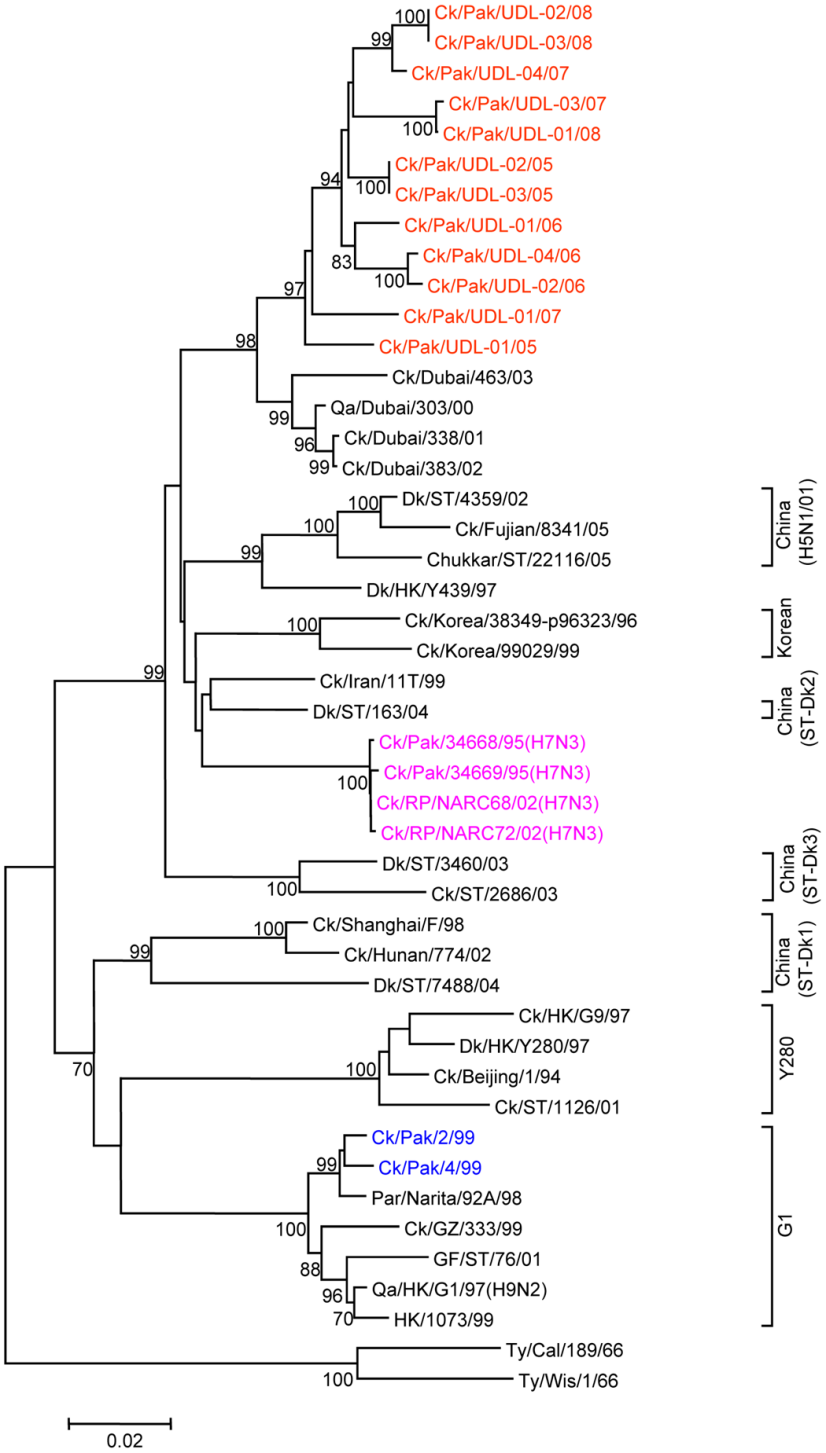
Phylogenetic relationships of the PA genes. The phylogenetic methods and abbreviations were as described for figure 1.

To determine the genetic relationship of H9N2 avian influenza viruses currently prevalent in poultry in Pakistan, we selected twelve H9N2 viruses isolated between May 2005 and March 2008 from different districts of the Punjab and the NWFP of Pakistan, Supplementary Table 1. These districts have a large concentration of commercial poultry farms and a number of H5N1 and H7N3 outbreaks have also been reported in this region over the last few years (18). The H9N2 isolates were all defined as LPAI viruses by an intravenous pathogenicity test of diluted infectious allantoic fluid (Table 1). Complete full length sequencing of all 8 segments was performed on at least 5 cDNA clones for each segment and a consensus sequence for each virus was produced; there was only very limited polymorphism in the analysed sequences. Phylogenetic relationships were examined between these viruses and representative H9N2 viruses from Asia and the Middle East, along with the established Eurasian H9N2 lineages: namely the G1-lineage, the Y280-lineage and the Korean-lineage represented by prototype viruses A/Quail/Hong Kong/G1/97, A/Duck/Hong Kong/Y280/97 and A/Chicken/Korea/38349-p96323/96 respectively, and with viruses identified in the Far East as emerging H9N2 lineages [6], [25]. Phylogenetic analysis of the HA revealed that all twelve isolates cluster together with G1-lineage viruses and show a very close relationship (93.0 to 96.4% nucleotide identity) with recent poultry-isolated H9N2 viruses from Iran (Figure 1 and Table 2), which shares a border with Pakistan. A slightly higher divergence (6.3 to 9.7%) was seen between HA genes of H9N2 viruses isolated from Pakistan in 1999 (Ck/Pakistan/2/99, Ck/Pakistan/4/99, and Ck/Pakistan/5/99); another set of viruses from Middle East isolated between 2000 and 2003 [14] also cluster with this group (Figure 1). The phylogenetic relationship of NA and M genes of the Pakistan viruses also fell within the G1-lineage and showed close identity (93.0–97.0% in the NA gene) with Ck/Pakistan/2/99, Ck/Pakistan/4/99, and Ck/Pakistan/5/99 (Figure 2, 3 and Table 2) and for the M gene 96.5 to 97.6% nucleotide identity with A/Ck/Pakistan/2/99. Like the HA, NA and M genes, the NP genes also grouped together with G1-lineage viruses (Figure 4): the closest nucleotide sequence identity (95.8–97.3%, Table 2) was found with viruses isolated from Pakistan during 1999 and A/Parakeet/Chiba/1/97, an introduction thought to be imported from Pakistan [26]. There was relatively little nucleotide divergence (2.4–7.0%) of NA, M and NP gene compared with H9N2 viruses isolated from Pakistan in 1999 (Ck/Pakistan/2/99, Ck/Pakistan/4/99 and Ck/Pakistan/5/99), which were of the G1-lineage but within this lineage some sub-clustering is evident (Figure 1, 2, 3 and 4). The phylogenetic analyses of each of the three polymerase complex genes (PB2, PB1 and PA), in contrast to the HA, NA, M and the NP genes, did not branch with exemplars of the G1-lineage or any of the other established Eurasian lineages (Figure 5, 6 and 7). All three polymerase complex genes were most closely related to those of H9N2 viruses isolated from the Persian Gulf and India between 2000 and 2004 [14], [27]. Nucleotide identities were from 94.1 to 96.4% for the PA gene, 91.2–94.4% for the PB1 gene and 94.5–96.6% identity in PB2; these similarities may indicate a separate Indian sub-continental lineage of H9N2 virus [6], [25]. This notion is supported by conclusions based on analyses of H9N2 viruses from the Middle East [14] where it was observed that reassortment of the PB2 gene had taken place and it was postulated that a UAE lineage of H9N2 viruses may have emerged.

**Table 1:** Comparison of IVPI and amino acid residues in several key sites of HA, NA and NS1 proteins.

Virus strain	IVPI	HA						NA						NS1		
		Receptor binding site				Cleavage Site	Glycosylation site at position	Presence of NA stalk deletion			HB site			Deletion of aa	Total no of aa	PL motif
		191 (183)^a^	198 (190)^a^	234 (226)^a^	235 (227)^a^	335–338	218 (200)^a^	38–39	46–50	62–64	372	402	403	80–84		
A/Chicken/Pakistan/2/99	ND	H	A	Q	Q	RSSR	Yes	No	No	No	T	N	W	No	230	EPEV
A/Quail/Hong Kong/G1/97	ND	H	E	L	Q	RSSR	Yes	Yes	No	No	S	I	R	No	230	EPEV
A/Hong Kong/1073/99	ND	H	E	L	Q	RSSR	Yes	Yes	No	No	S	N	W	No		EPEV
A/Duck/Hong Kong/Y280/97	ND	N	T	L	Q	RSSR	Yes	No	No	Yes	S	N	W	No	230	EPEV
A/Chicken/Korea/99029/99	ND	H	E	Q	Q	ASGR	Yes	No	No	No	S	N	W	No	230	ESEV
A/Chicken/Pakistan/UDL-01/05	0.00	H	A	L	I	RSSR	No	No	No	No	A	N	W	Yes	225	ESKV
A/Chicken/Pakistan/UDL-02/05	0.00	H	A	L	I	RSSR	No	No	Yes	No	A	N	R	Yes	225	ESKV
A/Chicken/Pakistan/UDL-03/05	0.00	H	A	L	I	RSSR	No	No	Yes	No	A	N	R	Yes	225	ESKV
A/Chicken/Pakistan/UDL-01/06	0.02	H	A	L	I	RSSR	No	No	No	No	A	N	R	No	217	LPPK
A/Chicken/Pakistan/UDL-02/06	0.00	H	A	L	I	RSSR	No	No	No	No	A	N	R	No	230	KSEI
A/Chicken/Pakistan/UDL-04/06	0.00	H	A	L	I	RSSR	No	No	No	No	A	S	R	Yes	225	ESKV
A/Chicken/Pakistan/UDL-01/07	0.00	H	A	L	I	RSSR	No	No	No	No	A	N	R	No	230	KSEI
A/Chicken/Pakistan/UDL-03/07	0.01	H	A	L	I	KSSR	No	No	No	No	A	N	R	Yes	225	ESKV
A/Chicken/Pakistan/UDL-04/07	0.03	H	A	L	I	RSSR	No	No	No	No	T	N	R	No	230	KSEI
A/Chicken/Pakistan/UDL-01/08	0.00	H	A	L	I	KSSR	No	No	No	No	A	N	R	No	230	KSEI
A/Chicken/Pakistan/UDL-02/08	0.01	H	A	L	I	KSSR	No	No	No	No	A	N	R	No	230	KSEI
A/Chicken/Pakistan/UDL-03/08	0.01	H	A	L	I	KSSR	No	No	No	No	A	N	R	No	230	KSEI

a Numbering according to H3 in parentheses, IVPI (intravenous pathogenicity index), HB (hemadsorbing site), aa (amino acids) PL (PL motif is referred as PDZ-binding motif at the C-terminal end of the NS1 protein). ND not determined in this study.

**Table 2:** Nucleotide similarities (%*) between H9N2 viruses isolated in Pakistan during 2005–2008 and other influenza viruses.

Gene	Viruses showing highest similarity	Identity (%)
PB2	A/Quail/Dubai/303/00 (H9N2)	95.3–96.6
PB1	A/Chicken/Dubai/463/03 (H9N2)	93.1–94.2
PA	A/Quail/Dubai/303/00 (H9N2)	95.2–96.4
HA	A/Chicken/Iran/B102/05 (H9N2)	93.9–96.4
NP	A/Parakeet/Chiba/1/97 (H9N2)	96.0–97.3
NA	A/Chicken/Pakistan/2/99 (H9N2)	93.0–97.0
M	A/Chicken/Pakistan/2/99 (H9N2)	96.5–97.6

* Identities were calculated based on complete open reading frames of each RNA segment for all twelve virus isolates sequenced in this study.

**Figure 8:**
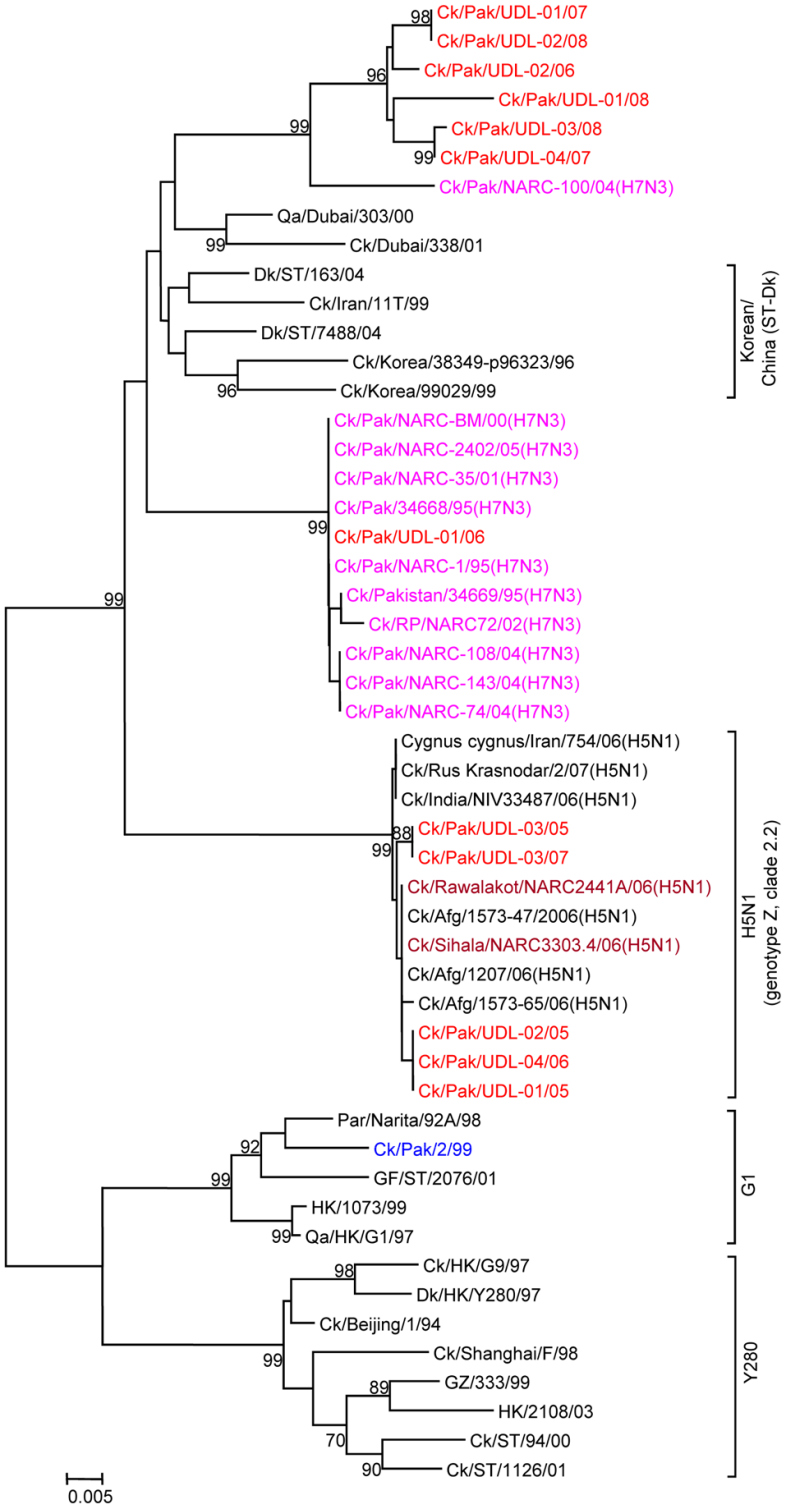
Phylogenetic relationships of the NS genes of H9N2 avian influenza viruses isolated from poultry in Pakistan. Analysis was based on nucleotides 27–870 of segment eight of all viruses included in this analysis. NS gene sequences were compared with closely related H5, H7 and H9 viruses (the viruses highlighted in red were sequenced in this study and H9N2, H7N3 and H5N1 viruses isolated in Pakistan previously are indicated in blue, pink and brown respectively). The phylogenetic methods and abbreviations were as described for figure 1.

In contrast to the other segments phylogenetic analysis of the NS gene segment of UDL viruses split the 12 viruses into three distinct groups (Figure 8). Six isolates of one group cluster together with 97.8% to 100% identity to each other and showed closest nucleotide identity (95.9–96.6%) with Ck/Pak/NARC-100/04, an H7N3 virus (Figure 8 and Table 3). The second group (represented by a single virus, A/Ck/Pak/UDL-01/06) showed a strikingly close relationship with 99.8 to 100% nucleotide identity within the NS1 coding region to H7N3 viruses isolated from Pakistan between 1995 and 2005. Members of this group contained a 13 amino acid deletion at the C-terminus of NS1 and this group was quite distinct from the Ck/Pak/NARC-100/04, H7N3 - group of viruses. The third group of five H9N2 viruses showed highest nucleotide similarity (99.3–99.7%) with Ck/Rawalakot/NARC2441A/06 and Ck/Sihala/NARC3033.4/2006, Ck/Afghanistan/1207/06 and Ck/Afghanistan/1573-47/06 (H5N1) viruses (Table 3) and clustered together with Clade 2.2 highly pathogenic H5N1 viruses of the Z-genotype (Figure 8), the closest related H5N1 viruses being isolated in Pakistan, India, Iran and Russia during 2006 [28], [29]. These five viruses were able to be differentiated into two groups, but both groups were very similar to other viruses isolated in the region.

**Table 3:** Nucleotide similarities (%) of the NS genes between H9N2 viruses isolated in Pakistan during 2005–2008 and NS genes of other influenza viruses.

H9N2 viruses isolated from Pakistan	Viruses in the database showing highest similarity	Identity (%)
A/Chicken/Pakistan/UDL-02/06	A/Chicken/Pakistan/NARC-100/04 (H7N3)*	95.9 to 96.7
A/Chicken/Pakistan/UDL-01/07		
A/Chicken/Pakistan/UDL-04/07		
A/Chicken/Pakistan/UDL-01/08		
A/Chicken/Pakistan/UDL-02/08		
A/Chicken/Pakistan/UDL-03/08		
A/Chicken/Pakistan/UDL-01/06	A/Chicken/Pakistan/34668/95 (H7N3)*	99.5 to 100
	A/Chicken/Pakistan/NARC-1/95 (H7N3)*	
	A/Chicken/Pakistan/NARC-143/04 (H7N3)*	
	A/Chicken/Pakistan/NARC-2402/05 (H7N3)*	
	A/Chicken/Pakistan/NARC-74/04 (H7N3)*	
	A/Chicken/Pakistan/NARC-BM/00 (H7N3)*	
	A/Chicken/Pakistan/NARC-108/04 (H7N3)*	
	A/Chicken/Pakistan/NARC-35/01 (H7N3)*	
A/Chicken/Pakistan/UDL-01/05	A/Chicken/Afghanistan/1207/06 (H5N1)	99.5 to 99.7
A/Chicken/Pakistan/UDL-02/05	A/Chicken/Afghanistan/1573-47/06 (H5N1)	
A/Chicken/Pakistan/UDL-03/05	A/Chicken/India/NIV33487/06 (H5N1)	
A/Chicken/Pakistan/UDL-04/06	A/Cygnus cygnus/Iran/754/06 (H5N1)	
A/Chicken/Pakistan/UDL-03/07	A/Chicken/Rawalakot/NARC2441A/06(H5N1)	
	A/Chicken/Sihala/NARC3303.4/2006(H5N1)	

* NS1 sequences only available in public databases, therefore percentage identities were calculated based on the NS1 coding region nucleotide sequences.

**Figure 9:**
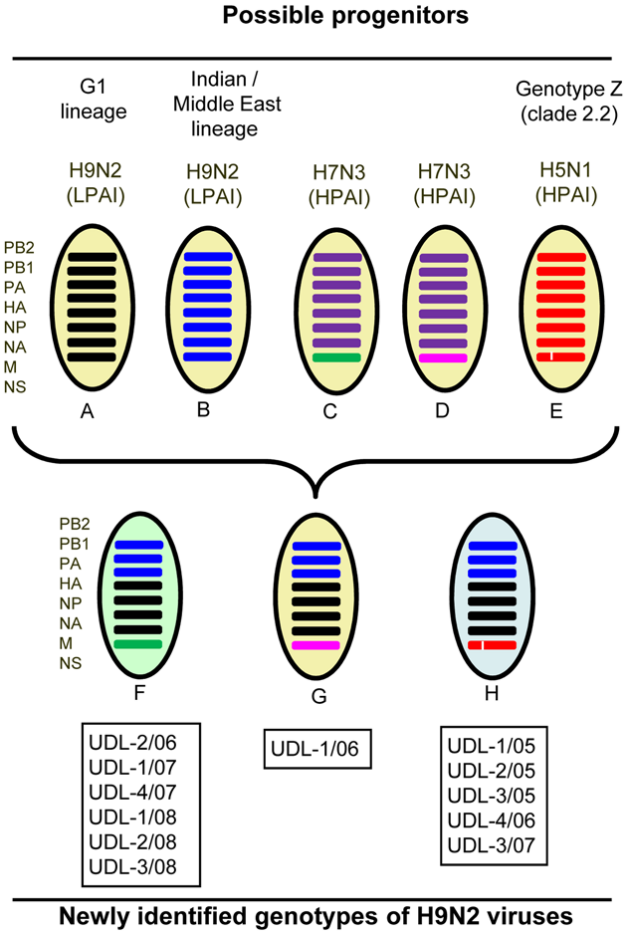
Representation of the newly identified genotypes of H9N2 viruses in poultry in Pakistan. The possible genetic progenitors of these new genotypes are (A) H9N2 (LPAI) viruses represented by A/Ck/Pak/2/99, belonging to G1-lineage, (B) H9N2 (LPAI) viruses represented as Indian/Middle East lineage containing distinct polymerase complex genes, (C) H7N3 (HPAI) viruses represented by the prototype A/Ck/Pak/NARC-100/2004 virus, (D) H7N3 (HPAI) viruses represented by the prototype A/Ck/Pak/34668/95 virus containing a 13 amino acid deletion in their NS1 polypeptide and (E) H5N1 (HPAI) viruses represented by A/Ck/Afghanistan/1207/2006 belonging to genotype Z. The three newly identified H9N2 genotypes (F, G and H) contained PB2, PB1, and PA genes similar to an Indian/Middle East lineage of H9N2 virus, along with the NS genes derived from two distinct H7N3 parental viruses and an H5N1 parental virus and the HA, NA, NP and M genes from H9N2 viruses of the G1 lineage.

The pattern of nucleotide similarity indicates that the 12 H9N2 viruses retained 4 genes (HA, M, NA and NP) similar to the H9N2 G1-lineage, 3 genes (PB1, PB2, PA) from a lineage of viruses from the Indian sub-continent and the Middle-East, and NS genes from one of two H7N3 lineages or from H5N1 genotype Z viruses that were previously present in Pakistan and neighbouring countries (Figure 9). A set of viruses from the Middle East studied by Aamir et al. [14] also showed evidence of reassortment of the NS gene segment in the proposed UAE lineage and it is possible that the UAE viruses and the six viruses from Pakistan that are closest to the Ck/Pak/NARC-100/04 H7N3 virus may share a common ancestor. Although the HA and NA gene sequences have drifted over the years (Figure S1), the H9N2 lineage-defining residues [17], [25] have been conserved: eight amino acids in HA (L^17^, T^96^, V^179^, L^209^, G^262^, S^290^, T^304^ in HA1 and V^91^ in HA2); seven amino acids ( L^10^, T^43^, S^77^, S^153^, T^212^, V^307^, G^346^) in NA. Similarly, M and NP genes also retained conserved G1-lineage defining residues (A^157^) in M1, one (L^10^) in M2, and four amino acids (Q^52^, V^317^, A^374^, K^430^) in NP.

### Molecular characteristics

In order to identify molecular markers that may correlate with the pathogenic properties of these H9N2 UDL isolates analysis was performed to compare deduced amino acid sequences of the envelope glycoproteins and the internal proteins of the Pakistan virus isolates with representative strains of established H9N2 virus Eurasian lineages.

### Hemagglutinin

Changes in the HA glycoprotein are central to the switch from LPAI to HPAI, but additional amino acid substitutions in the HA molecule have been associated with inter-species adaptation [30]. The key molecular determinants of pathogenicity and virulence in the HA molecule are the HA1/HA2 connecting peptide sequence, specific amino acids residues at the receptor binding site, and the presence or absence of glycosylation sites near the receptor binding site [31]. As expected from the pathogenicity tests, nucleotide sequence analysis corresponding to the HA1/HA2 junction of these viruses revealed no poly-basic cleavage motif. Eight out of twelve isolates retained an R-S-S-R motif at the cleavage site which is the signature of H9N2 LPAI viruses adapted to the chicken host, as seen in recent years in H9N2 virus isolates from Asia and the Middle East [14], [25], [26], [27], [32], [33]. It was noted that four UDL isolates possess lysine instead of arginine at the −4 position of the cleavage site (Table 1). Lysine at this position has been observed only infrequently in H9N2 viruses [34] and the significance, if any, of this alteration from one basic amino acid to another is not known.

The receptor binding site motif of HA is critical for cellular receptor specificity and determining virus host range [35], [36]. Residues at positions 183, 190, 226, 227 and 228 (H3 numbering) are major components of the receptor-binding site of the HA molecule. All UDL viruses showed conservation of residues H^183^, A^190^, L ^226^, I^227^ and G^228^ in the receptor binding cleft. Of these, three represent substitutions (E/T^190^A, Q^226^L and Q^227^I) when compared with the reference strains of the H9 lineage (Table 1). Glutamine at position 226 dictates a preferential binding to SA α2,3-linked to galactose found in avian hosts and is a major host range determinant for influenza A viruses of the H2 and the H3 subtypes associated with pandemic human infections [37], [38]. The substitution, as seen in the viruses under study here, of Q^226^L at the receptor binding site in the HA allows H9N2 viruses to replicate more efficiently (with 100-fold higher peak titres) in human cells in culture [39], [40] and is associated with a preferential receptor binding specificity for sialic acid α2,6-linked galactose [37] and was also observed in the UAE isolates analysed by Aamir [14]. The significance of E→A and Q→I substitutions at positions 190 and 227 respectively observed in all UDL isolates on the receptor binding specificity within this group of viruses is not known and requires detailed structural analysis to resolve their importance. In addition, the alteration at position 150 (138 in H3) from Ala to Ser, which may affect the conformation of residues lining the receptor binding site, has also been linked to changes in receptor specificity in H1 viruses [38]. Analysis of potential glycosylation site motifs N-X-S/T in the HA molecule of the UDL viruses revealed seven sites at positions 29, 105, 141, 298, 305, 492 and at position 551, C-terminal to the membrane anchor (21, 94, 132, 289, and 296 in HA1 and 154 and 213 in HA2 in H3 numbering). One potential glycosylation site at position 218 (210 in H3) was lost compared with representative reference strains of the G1-lineage, the Y280-lineage, the Korean-lineage and the prototypic Pakistani isolate Ck/Pakistan/2/99 [41] (Table 1). Loss of an additional glycosylation site at position 206, compared with the prototype G1 virus A/Qa/HK/G1/97 was observed; this was also seen in H9N2 viruses from the Middle East in 2000–2003 [14]. The loss of potential glycosylation sites may represent a selected adaptation of H9N2 within poultry since alteration in glycosylation pattern has been suggested to influence adaptation of avian influenza viruses to poultry [30], [31].

### Neuraminidase

The major molecular determinants that are known to influence the functional activities of the neuraminidase (NA) glycoprotein are the enzyme active site, the stalk length, the sialic acid binding site also referred to as the hemadsorbing site (HB), and potential glycosylation sites. The neuraminidase sequences of all 12 isolates were compared with N2 NA sequences of H9N2 viruses prevalent in Asia and the Middle East during the last fifteen years. For each virus amino acids in the enzyme active site were conserved and showed no evidence of substitutions associated with resistance to the sialidase inhibitors oseltamivir and zanamivir. The data revealed that, of the 12 viruses analysed, 2 isolates (Ck/Pak/UDL-02/05 and Ck/Pak/UDL-03/05) contained a unique 5 amino acid residue deletion (aa 46–50) in the stalk region, the others having no deletion in the NA stalk, as was also observed in H9N2 viruses from the Middle East [14]. Deletions in the stalk of avian N2 viruses have been observed previously in H9N2 viruses: the two prototype viruses, Qa/HK/G1/97 and HK/1073/97, of the G1-lineage contained a two amino acid deletion in the NA stalk region at positions 38 and 39, and Dk/HK/Y280/97 and Ck/Shanghai/F/98, another virus belonging to the Y-280-lineage, lacked three amino acid at positions 63–65 in the NA stalk region (Table 1). The precise 5 amino acid deletion at position 46–50 in the stalk region represents a unique event not observed previously in H9N2 viruses. It is recognised that deletion within the stalk region of the neuraminidase may be an important feature balancing the complementary activities of the HA and NA on adaptation of virus to poultry [30], [31].

Analysis of the HB site, which is located on the surface of the NA molecule away from the neuraminidase enzyme active site at positions 366–373, Asn-400, Trp-403 and Lys-432 [42], [43], revealed that although these sites have been well conserved in H9N2 viruses isolated from wild aquatic birds [44], poultry-adapted H9N2 viruses, including isolates sequenced in this study, contained substitutions similar to those detected in both A/Qa/HK/G1/97 and A/Dk/HK/Y280/97. These substitutions were seen previously in H9N2 human isolates from Hong Kong in 1999 and are typical of human pandemic H2N2 and H3N2 viruses [45]. The substitutions at residue 372 from serine to alanine or, for one virus, threonine (Table 1) were similar to those detected in Parakeet/Narita/92A/98, Ck/Pak/2/99 and H9N2 viruses isolated from Iran and the United Arab Emirates [14], [26]. In addition, a substitution from tryptophan to arginine at position 403 was observed in 11 of the 12 viruses recently isolated from Pakistan; this substitution was present in Qa/HK/G1/97 but not in other H9N2 viruses, including those from the UAE from 2000 to 2003 [14] and, importantly, not in the human (e.g. A/Hong Kong/1073/99) H9N2 isolates (Table 1). Within H9N2 viruses, polymorphism is seen at this site with W, L, S or R present. The Qa/Hong Kong/G1/97 isolate is unusual in carrying a N402I substitution and one of the viruses characterised here (A/Ck/Pak/UDL-04/06) has serine at this position. The biological significance of any of these substitutions in the HB site is not known.

The comparison of conserved potential glycosylation sites in NA glycoprotein showed that the majority of H9N2 viruses belonging to established Eurasian lineages contain seven conserved potential glycosylation sites at positions 61, 69, 86, 146, 200, 234 and 402 (N2 numbering). The viruses sequenced in this study contained an additional glycosylation site at Asn 44 due to the substitution of Pro45Ser. This additional glycosylation site had also been reported in a number of other H9N2 viruses including Ck/Ind/2048/03, Ck/HK/G9/97 [27]. One UDL isolate Ck/Pak/UDL-02/06 lacked one potential glycosylation site at Asn 234 due to T^236^I substitution. Again, the significance of glycosylation changes at sites 44 and 234 is unknown; however, addition or loss of potential glycosylation may contribute towards increased virulence [46] due to altered antigenicity or sialidase activity.

A comparison of the amino acid sequences of the H9N2 neuraminidases shows that certain amino acid substitutions have become fixed as the viruses have evolved but many are unique substitutions represented in individual viruses (Supplementary Figure S1B). It appears that fewer amino acids were unique to individual viruses in the HA than in the NA; this may reflect different evolutionary pressures applied to the two glycoproteins. The necessity for compensatory changes in the NA to ensure its compatibility with the HA as the HA evolves may be one such force.

### NS1 and NS2 proteins

The NS gene showed a high level of sequence diversity among the twelve UDL isolates analysed. Of particular note were five isolates (UDL-01/05, UDL-02/05, UDL-03/05, UDL-04/06, UDL-03/07) that contained an NS gene closely related to the highly pathogenic H5N1 viruses belonging to clade 2.2 of genotype Z with over 99% nucleotide identity (Figure 8 and Table 3). The NS1 portions of these viruses showed between 0 and 3 amino acid changes from H5N1 viruses isolated during 2006 to 2007 from Pakistan, Afghanistan, Iran, India, and Russia [28] and their NS1 proteins, being from of the Z-lineage H5N1 viruses, had a five-amino acid deletion (deleting residues 80–84) resulting in an NS1 protein of 225 amino acids in length; the NS1 proteins also contained the “ESKV” PDZ ligand (PL) C-terminal motif typical of H5N1 viruses of the Z-genotype (Table 1). The role of the NS1 protein in infection is complex and includes countering interferon and cytokine induction (reviewed recently in Hale et al. [47]) and its effects on pathogenesis have become clearer but the biological significance of the 5 amino acid deletion is not well understood. It has been reported that viruses containing NS1 with deleted (^80^TIASV^84^) residues show increased virulence in both mouse and poultry infections [48]. The specific functional role of this deletion in H9N2 viruses needs to be determined but there was no evidence of any increased pathogenicity associated with the deletion in our IVPI tests.

RNA segment 8 of one virus (UDL-01/06) was very closely related to that of H7N3 viruses from Pakistan isolated over an eleven year period. This single virus isolate shared a 13 amino acid C-terminal truncation; similar truncations have been reported previously in H7 and H9 subtype viruses isolated from poultry [49], [50], suggesting a natural virus adaptation, but the significance of this truncation and the resulting C-terminal LPPK motif to the virus life cycle is also not understood.

The other 6 viruses (UDL-02/06, UDL-01/07, UDL-04/07, UDL-01/08, UDL-02/08, and UDL-03/08) formed a distinct sub-clade and showed no truncation or deletion. They contained a PL motif “KSEI”. This KSEI sequence as a PL motif is uncommon. The large scale sequence analysis of avian influenza viruses reported by Obenauer et al. [29] revealed that a C-terminal isoleucine residue in NS1 was rare: out of 1196 PL motif sequences only 48 sequences from avian-origin viruses and one from a swine-origin virus contained I at the C-terminus. In addition, K at the −4 position in the PL motif was also rare, with the H1N1 1918 pandemic virus, two H5N1 viruses isolated during 2005 in Indonesia and a further two H5N1 viruses isolated in 2007 from Saudi Arabia which contained a “KSEV” C-terminal sequence in the NS1 protein, being the only examples reported [29], [51]. We can summarise, the H9N2 viruses circulating recently in Pakistan have three distinct C-terminal motifs in NS1: KSEI, ESKV and LPPK. The importance of the C-terminal residues of NS1 has been demonstrated recently in mouse studies, which showed that the insertion of four C-terminal amino acids, either ESEV, EPEV, or KSEV, into otherwise avirulent viruses resulted in an increase in virus virulence and caused severe disease signs [52]. However, it is not known whether deletion or variation in the PL motif of NS1 proteins influence the virulence of H9N2 viruses in the poultry host.

### Nucleoprotein and polymerase proteins

A number of residues in the polymerase proteins (PB1, PB2 and PA) and in the nucleoprotein (NP) are known to play a key role in the host range of AI viruses to increase virulence or replication in the mammalian host. Some may be considered as hall-marks of avian or mammalian viruses, whilst others can influence replication efficiency in mammalian or avian hosts. Two analyses of molecular changes associated with the transmission of avian-origin H5N1 and H9N2 viruses to humans [53], [54] showed that the PB2, PA and NP proteins contained a number of distinct host-specific residues (Table S2). Of the 44 host-associated genetic signatures of avian- and human-host origin viruses, 42 residues were identical in all 12 UDL isolates and show the avian host signature. The exceptions were that a single isolate (Ck/Pak/UDL-04/06) contained a S^44^A substitution in PB2 and all isolates had aspartic acid at position 372 in NP which has been found in avian influenza viruses isolated from humans [54].

Additional residues in the ribonucleoproteins implicated in enhanced replication in mammalian cells have been defined (Table S2) and these residues have been examined in the UDL H9N2 viruses under study here. These viruses all possessed the typical avian residue at each of these locations excepting positions 13 in PB1 (Proline not Leucine was present) and residue 34 in the NP protein, which was Glycine and not Asparagine, the mouse preferred counterpart, or the more typical avian residue Aspartic acid. Proline at position 13 of PB1 is common within H9N2 viruses.

RNA segment 2 encodes a second polypeptide in addition to PB1, termed PB1-F2 [55]. The understanding of the importance of PB1-F2 in virus pathogenicity in mouse models has increased recently [56], [57], [58] and the observation that over 95% of avian influenza viruses encode a full length PB1-F2 [59] highlights the potential importance of the PB1-F2 in the avian host. It is striking that N^66^S in PB1-F2 is associated with increased virulence in a mouse model [57] but viruses having S at residue 66 of PB1-F2 are rare and were not present in the H9N2 viruses in this study. Table S2 lists the amino acid substitutions associated with host range for the genes encoding polypeptides of the replication complex and the PB1-F2 polypeptide.

It is notable that the three polymerase polypeptides have reassorted as a group and the maintenance of the avian specificity at residue 627 of PB2 is striking. The reason for the maintenance of the three polymerase polypeptides may be chance or it is quite likely that the three PB1, PB2 and PA polypeptides act most efficiently as a set and so there may be pressure to maintain a gene constellation for optimal replication proficiency. This may be analogous to the situation observed in human influenza A viruses in which H1N1 and H3N2 co-circulate but with only very limited gene reassortment [60], [61], [62]. In contrast though, the results of an analysis of gene reassortment in a wide variety of avian influenza isolates showed that apparently free reassortment among the genes encoding the virus replication complex was on-going [63].

### M1 and M2 proteins

Several amino acids in the virus matrix, M1, protein are linked with increased replication in mammals or increased pathogenicity in small animal models (Table S2). At residue 15, all the H9N2 UDL viruses encode isoleucine and the amino acid substitution of V^15^I is common within H9N2 lineages. The UDL viruses showed heterogeneity at residue 37 with 5 viruses encoding valine and 7 viruses alanine but the substitution T^37^A/V clusters exclusively with the recent UDL H9N2 viruses from Pakistan. The significance of any of these changes to the potential of the currently evolving H9N2 viruses in Pakistan to increase their host range is not known.

None of the UDL viruses contained substitutions at amino acid positions 26, 27, 30, 31 or 34 in the M2 proteins suggesting that these viruses have no resistance to amantadine [64], [65] which contrasts with viruses from the Middle East isolated between 2000 and 2003 [14]. Residues associated with host range have been adduced for M2, again summarised in Table S2. The recent H9N2 viruses show the typical avian sequence at several of the sites but at residue 11, two virus isolates encoded isoleucine; at residue 16 nine of the twelve isolates encoded glycine whilst three encoded aspartic acid; at residue 20 lysine was present; at position 28 isolates contained isoleucine or valine – the majority valine; and residue 55 was phenylalanine. Whether these changes result in increased replication efficiency in mammalian cells has to be determined.

### Conclusion

The emergence of these novel genotypes of H9N2 viruses and the sustained prevalence of these viruses in poultry warrant further surveillance of H9N2 viruses by complete genomic analysis. These viruses are endemic in poultry in many countries in Asia and the surrounding regions and the generation of new genotypes within H9N2 viruses by reassortment should not be ignored since it allows for an increased ability of the virus to adapt rapidly to different challenges. The observation shown here that further gene reassortment has occurred subsequent to the emergence of viruses in the Middle East highlights the potential for viruses to evolve rapidly. It is widely recognised that genetic reassortment in avian influenza viruses is common in wild bird and poultry isolates [63], [66], [67] but the species in which reassortment most readily occurs is not known for certain. The continuous circulation of H9N2 in chickens might suggest that reassortment may occur in domesticated poultry. Recent evidence links passage of H9N2 viruses in domestic poultry with increased virus replication in mouse models [68]. This observation combined with evidence of human and animal infection by H9N2 viruses [8], [9], [69], [70], [71], combined with the frequent exposure of people to infected poultry, argues that a greater effort should be put in place to manage and control not only H5 and H7 HPAI infections but also H9N2 virus infections in poultry to reduce the risk of a human influenza pandemic.

## Supporting Information

Figure S1 Amino acid changes in the surface glycoproteins of H9N2 viruses isolated from poultry in Pakistan between 2005 and 2008. The substitutions in the HA (A) and NA (B) are shown in coloured boxes and arrows represent the common changes at a particular node of the tree and the unique changes within individual isolates are represented by black boxes/arrows. Amino acid numbers are based on the H9 amino acid sequence starting at the initiator methionine residue.
(2.41 MB TIF)

Table S1 H9N2 viruses isolated from chicken flocks in Pakistan.
(0.06 MB DOC)

Table S2 Analysis of host range and pathogenicity determinants in the PB2, PB1, PA, NP, M1 and M2 proteins in H9N2 viruses isolated from poultry in Pakistan.
(0.38 MB DOC)
