# Supplementary material for: Novel Genotypes of H9N2 Influenza A Viruses Isolated from Poultry in Pakistan Containing NS Genes Similar to Highly Pathogenic H7N3 and H5N1 Viruses
Source: PLoS One. 2009 Jun 11;4(6):e5788. doi: 10.1371/journal.pone.0005788 (PMC2690689; doi:10.1371/journal.pone.0005788)
Supplement: Table S1 — H9N2 viruses isolated from chicken flocks in Pakistan. (0.06 MB DOC) [file pone.0005788.s002.doc]

**Table S1**. H9N2 viruses isolated from chicken flocks in Pakistan.

| **Isolates** | **Host** | **Collection Date** | **District** | **Region** |
| --- | --- | --- | --- | --- |
| A/chicken/Pakistan/UDL-01/2005 | Chicken (Breeder ) | 18-May-2005 | Sailkot | Punjab |
| A/chicken/Pakistan/UDL-02/2005 | Chicken | 31-Dec-2005 | Lahore | Punjab |
| A/chicken/Pakistan/UDL-03/2005 | Chicken (Broilers) | 21-Dec-2005 | Lahore-Raiwind | Punjab |
| A/chicken/Pakistan/UDL-01/2006 | Chicken (Breeder ) | 2-May-2006 | Peshawar | NWFP |
| A/chicken/Pakistan/UDL-02/2006 | Chicken (Broilers) | 29-Dec-2006 | Lahore-Raiwind | Punjab |
| A/chicken/Pakistan/UDL-04/2006 | Chicken (Broilers) | 19-Jun-2006 | Lahore-Manga | Punjab |
| A/chicken/Pakistan/UDL-01/2007 | Chicken (Layer) | 7-Jun-2007 | Faisalabad | Punjab |
| A/chicken/Pakistan/UDL-03/2007 | Chicken (Broilers) | 17-Dec-2007 | Kasur | Punjab |
| A/chicken/Pakistan/UDL-04/2007 | Chicken (Broilers) | 16-Mar-2007 | Lahore | Punjab |
| A/chicken/Pakistan/UDL-01/2008 | Chicken (Breeder ) | 9-Jan-2008 | Lahore-Raiwind | Punjab |
| A/chicken/Pakistan/UDL-02/2008 | Chicken (Broilers) | 22-Feb-2008 | Kasur-Bhaipharu | Punjab |
| A/chicken/Pakistan/UDL-03/2008 | Chicken (Breeder ) | 4-Mar-2008 | Lahore-Raiwind | Punjab |
