# Supplementary material for: Novel Genotypes of H9N2 Influenza A Viruses Isolated from Poultry in Pakistan Containing NS Genes Similar to Highly Pathogenic H7N3 and H5N1 Viruses
Source: PLoS One. 2009 Jun 11;4(6):e5788. doi: 10.1371/journal.pone.0005788 (PMC2690689; doi:10.1371/journal.pone.0005788)
Supplement: Table S2 — Analysis of host range and pathogenicity determinants in the PB2, PB1, PA, NP, M1 and M2 proteins in H9N2 viruses isolated from poultry in Pakistan. (0.38 MB DOC) [file pone.0005788.s003.doc]

**Table S2:** Analysis of host range and pathogenicity determinants in the PB2, PB1, PA, NP, M1 and M2 proteins in H9N2 viruses isolated from poultry in Pakistan.

| **Protein** | **Amino acids** | **UDL-H9N2^a^** | **Avian^b^** | **Mammalian^c^** | **Reference** |
| --- | --- | --- | --- | --- | --- |
| **PB2** | 44 | A (11) S(1) | A | S | [53,54] |
|  | 81 | T | T | M | [53] |
|  | 199 | A | A | S | [53,54] |
|  | 271 | T | T | A | [53,54] |
|  | *256* | *D* | *D* | *G* | [72] |
|  | *333* | *T* | *T* | *I* | [73] |
|  | *355* | *R* | *K* | *Q* | [74] |
|  | 475 | L | L | M | [54] |
|  | *482* | *K* | *K* | *R* | [73] |
|  | 588 | A | A | I | [53,54] |
|  | 613 | V | V | T | [53,54] |
|  | *627* | *E* | *E* | *K* | [54,73] |
|  | 661 | A | A | T | [53] |
|  | 674 | A | A/S | T | [53,54] |
|  | *701* | *D* | *D* | *N* | [73,75,76] |
|  | 702 | K | K | R | [53] |
|  | *714* | *S* | *S* | *R* | [73] |
| **PB1** | *13* | ***P*** | *L* | *P* | [73] |
|  | 327 | R | R | K | [54] |
|  | 336 | V | V | 1 | [54] |
|  | *538* | *D* | *D* | *G* | [73] |
|  | *578* | *K* | *K* | *Q* | [73] |
|  | *678* | *S* | *S* | *N* | [73] |
| **PB1-F2** | 73 | K | K | R | [54] |
|  | 76 | V | V | A | [54] |
|  | 79 | R(10) L(2) | R | Q | [54] |
|  | 82 | L | L | S | [54] |
|  | 87 | E(10) **G**(2) | E | G | [54] |
| **PA** | 28 | P | P | L | [53,54] |
|  | 55 | D | D | N | [53,54] |
|  | 57 | R | R | Q | [54] |
|  | *65* | *S* | *S* | L/*Y* | [53,73] |
|  | 100 | V(10) I(2) | V | A | [53] |
|  | *133* | *E* | *E* | *G* | [73] |
|  | 241 | C | C | Y | [53] |
|  | 225 | S | S | C | [54] |
|  | 268 | L | L | I | [54] |
|  | 312 | K | K | R | [53] |
|  | 356 | K | K | R | [54] |
|  | 382 | E | E | D | [53,54] |
|  | 400 | S | Q/T/S | L | [53] |
|  | 404 | A | A | S | [54] |
|  | 409 | S | S | N | [53,54] |
|  | 552 | T | T | S | [53,54] |
|  | *556* | *Q* | *Q* | *R* | [73] |
|  | *615* | *K* | *K* | *N* | [73] |

Table S2: continued

| **Protein** | **Amino acids**  **_** | **UDL-H9N2^a^** | **Avian^b^** | **Mammalian^c^** | **Reference** |
| --- | --- | --- | --- | --- | --- |
| **NP** | 16 | G | G | D | [54] |
|  | 31 | R | R | K | [53] |
|  | 33 | V | V | I | [53,54] |
|  | *34* | *G* | *D* | *N* | [73] |
|  | 61 | I | I | L | [53,54] |
|  | 100 | R | R | V | [53,54] |
|  | 109 | I | I | V | [54] |
|  | 127 | E | E | D | [53] |
|  | 136 | I | L | M | [53] |
|  | 214 | R | R | K | [53,54] |
|  | 283 | L | L | P | [53,54] |
|  | *319* | *N* | *N* | *K* | [73,76] |
|  | 293 | R | R | K | [53,54] |
|  | 305 | R | R | K | [54] |
|  | 313 | F | F | Y | [53,54] |
|  | 357 | Q | Q | K | [54] |
|  | 372 | **D** | E | D | [54] |
|  | 375 | D | D | G/E | [53] |
|  | 422 | R | R | K | [54] |
|  | 442 | T | T | A | [54] |
|  | 455 | D | D | E | [54] |
|  | *480* | *D* | *D* | *N* | [73] |
| **M1** | *15* | ***I*** | *V* | *I* | [74] |
|  | 115 | V | V | I | [54] |
|  | 121 | T | T | A | [54] |
|  | 137 | T | T | A | [53] |
| **M2** | 11 | T(10) I(2) | T | I | [54] |
|  | 16 | **G(9)D(3)** | E | G/D | [53] |
|  | 20 | K | S | N | [53,54] |
|  | 28 | **V**(9) I(3) | I | I/V | [53] |
|  | 55 | **F** | L | F | [53] |
|  | 57 | Y | Y | H | [54] |
|  | 78 | Q | Q | K | [53] |
|  | 86 | V | V | A | [54] |

^a^Sequences of H9N2 viruses analysed in this study; ^b^Viruses isolated from avian hosts; ^c^Viruses isolated from mammalian hosts; Numbers in parentheses are the number of virus variants containing specific amino acid residues; Bold indicate the residues identical to those in influenza viruses isolated from humans. *Italic* indicates the residues correlate with enhanced replication and pathogenicity in mammalian host.
